# UN-YOLOv5s: A UAV-Based Aerial Photography Detection Algorithm

**DOI:** 10.3390/s23135907

**Published:** 2023-06-26

**Authors:** Junmei Guo, Xingchen Liu, Lingyun Bi, Haiying Liu, Haitong Lou

**Affiliations:** The School of Information and Automation Engineering, Qilu University of Technology (Shandong Academy of Sciences), Jinan 250353, China; gjm@qlu.edu.cn (J.G.); 10431220700@stu.qlu.edu.cn (X.L.); 10431210560@stu.qlu.edu.cn (L.B.); 10431210427@stu.qlu.edu.cn (H.L.)

**Keywords:** YOLOv5, artificial intelligence, target detection, aerial image

## Abstract

With the progress of science and technology, artificial intelligence is widely used in various disciplines and has produced amazing results. The research of the target detection algorithm has significantly improved the performance and role of unmanned aerial vehicles (UAVs), and plays an irreplaceable role in preventing forest fires, evacuating crowded people, surveying and rescuing explorers. At this stage, the target detection algorithm deployed in UAVs has been applied to production and life, but making the detection accuracy higher and better adaptability is still the motivation for researchers to continue to study. In aerial images, due to the high shooting height, small size, low resolution and few features, it is difficult to be detected by conventional target detection algorithms. In this paper, the UN-YOLOv5s algorithm can solve the difficult problem of small target detection excellently. The more accurate small target detection (MASD) mechanism is used to greatly improve the detection accuracy of small and medium targets, The multi-scale feature fusion (MCF) path is combined to fuse the semantic information and location information of the image to improve the expression ability of the novel model. The new convolution SimAM residual (CSR) module is introduced to make the network more stable and focused. On the VisDrone dataset, the mean average precision (mAP) of UAV necessity you only look once v5s(UN-YOLOv5s) is 8.4% higher than that of the original algorithm. Compared with the same version, YOLOv5l, the mAP is increased by 2.2%, and the Giga Floating-point Operations Per Second (GFLOPs) is reduced by 65.3%. Compared with the same series of YOLOv3, the mAP is increased by 1.8%, and GFLOPs is reduced by 75.8%. Compared with the same series of YOLOv8s, the detection accuracy of the mAP is improved by 1.1%.

## 1. Introduction

With the rapid development of UAVs [1], the application scenarios of the UAV industry are more and more rich. In aerial photography, agricultural plant protection, public security, UAV mapping, disaster rescue, inspection service and other scenes, UAVs can complete the task needs in real time and effectively, which is convenient for people management, monitoring and rescue. The current UAV has the advantages of convenient carrying, simple operation, rich load and rapid response, but also has the problems of short flight time and low detection accuracy. How to improve the detection accuracy of UAV aerial photography is the main challenge faced by researchers. At present, a large number of researchers have carried out research on the detection performance of UAVs. For example, Syed Samiul Alam et al. proposed RF-supported deep learning-assisted UAV detection and identification [2]. Athanasios Tsoukalas et al. proposed tracking evasive drones with deep learning assistance [3]. Sungtae Moon et al. proposed realizing real-time monitoring of large areas on drones through image stitching [4]. H.t. Lou et al. used DC-YOLOv8 to improve the performance of small object detection [5]. Researchers continue to explore and practice.

Benefiting from the common progress of algorithms, data and computing power, artificial intelligence [6] technology is currently in the third peak of development. Today, with the collision of science and technology and thinking, science and technology are developing rapidly and integrating into human production and life. It greatly improves work efficiency, reduces the cost of production and management, injects power into the development of economy and increases enthusiasm into the development of scientific research. Object detection [7] is one of the core problems in the field of computer vision. At present, it is mainly divided into two categories: one stage and two stage. The one-stage network is able to predict the category and location of the object while generating candidate regions. It has fast detection speed and strong generalization.The classic algorithms are SSD [8], Retina-Net [9], and YOLO family [10,11,12,13,14,15]. The two-stage network is performed in two steps: firstly, the candidate regions are generated, and then the target is predicted and classified by the convolutional neural network. It has high accuracy but a long training time. The more classical algorithms are RCNN series [16,17,18,19], R-FCN [20], and SPPNet [21].

Compared with the other algorithms, YOLOv5 belongs to the one-stage object detection method, which plays an excellent role in various fields of society. The main idea is to divide the whole image into several grids. Each grid predicts the type and location information of the objects in that grid. Then, the target box is filtered according to the IoU [22] value between the predicted box and the true box. Finally, the category and location information of the prediction box are output. YOLOv5 uses mechanisms such as multi-scale prediction and CIoU loss to improve the detection accuracy. At the same time, it uses Anchor detection to improve the detection speed. The model can be compiled to ONNX and CoreML, which has great advantages in rapid deployment. The international research on YOLOv5 series algorithms has never stopped. For example, Chenyang Wei et al. proposed rapid detection of helmets and license plates based on an improved YOLOv5 [23]. Xuehu Duan et al. put forward the DBF-YOLO algorithm to improve the accuracy of small target detection [24]. Wentao Zhao et al. came up with CR-YOLOv5s to detect the flowering time of chrysanthemums [25], and so on. Researchers are enthusiastic about YOLOv5.

Based on the consideration of UAV deployment application and the generalization detection of small target objects, this paper proposed a novel improved algorithm based on YOLOv5s named UN-YOLOv5s. The main contributions of the proposed algorithm are as follows:

(1) In order to solve the problem of gradient disappearance or explosion and equal distribution of attention weights caused by the deepening of the model network, a CSR module is set to make the stable transmission of feature information and always pay attention to effective information.
(2) Aiming at the problem of missing location information caused by continuous convolution, feature fusion is used to fuse deep semantic information and shallow location information to improve the generalization ability of the model.
(3) Increase the size of the detection head by up-sampling twice to obtain a smaller feature prediction map, improving prediction probability. At the same time, a more suitable anchor box is selected to improve the detection accuracy.

## 2. Related Work

### 2.1. Introduction to the YOLO Algorithm

YOLO [10,11,12] is an object detection model proposed by Joseph Redmon and Ali Farhadi et al. in 2015. It stands for you only look once (YOLO), and it’s a one-stage detection algorithm. It only takes one scan to identify the type and location of the objects in the image. After countless researchers transform and innovate the YOLO series algorithm, the YOLO series was developed and got continuous progress. Auxiliary techniques were introduced such as data augmentation [26] and loss functions [27], and has been updated to the eighth generation. Each generation of YOLO has its own advantages and disadvantages depending on the use case. Based on the deployment of UAVs and the maturity of the algorithm, we chose the YOLOv5 version for improvement.

Depending on the difference between depth multiple (dm) and width multiple (wm), YOLOv5 comes in four versions, YOLOv5s (dm = 0.33, wm = 0.50) is the minimal network model, YOLOv5m (dm = 0.67, wm = 0.75) is the medium network model, YOLOv5l (dm = 1.0,wm = 1.0) is the large network model, and YOLOv5x (dm = 1.33, wm = 1.25) is the largest network model.

### 2.2. YOLOv5 Loss Calculation

The loss [27] calculation of YOLOv5 is mainly composed of three parts:

Classes loss: BCE loss is adopted and only the classification loss of positive samples is calculated.

Objectness loss: BCE loss is adopted, and obj loss is calculated for all samples, where obj refers to the CIoU of the target bounding box predicted by the network versus the GT Box.

Location loss: CIoU loss is adopted and only the location loss of positive samples is calculated.

(1) $$L\mathrm{oss}=\lambda_{1}L_{\mathrm{cls}}+\lambda_{2}L_{\mathrm{obj}}+\lambda_{3}L_{\mathrm{loc}}$$

*L*_OSS_ is the total loss function of the model, $L_{\mathrm{cls}}$ signifies the value of the classification loss, $L_{\mathrm{obj}}$ presents the value of the confidence loss, $L_{\mathrm{loc}}$ denotes the value of the localization loss. $\lambda_{1}$, $\lambda_{2}$, $\lambda_{3}$ are the balance coefficients.

### 2.3. YOLOv5 Network Structure

**Figure 1:**
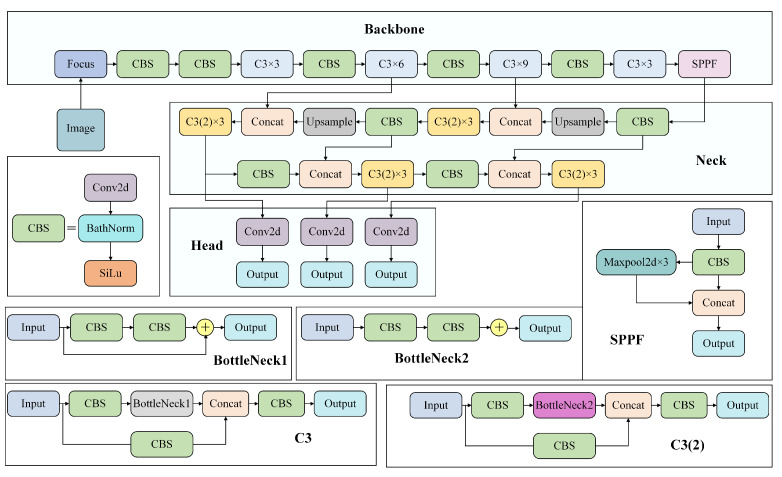
YOLOv5 network structure diagram.

The network structure of YOLOv5 is mainly composed of Backbone, Neck and Head parts. Backbone used Focus, Cross Stage Paritial Darknet53 (CSPDarknet53) and Spatial Pyramid Pooling–Fast (SPPF) modules. The CSPDarknet53 module improved the feature extraction ability of the model to a certain extent by using the residual structure and feature reuse mechanism and it prevented the gradient from disappearing and exploding. The SPPF module solved the multi-scale problem of the target well by fusing different sizes of MaxPool. The Neck part used Feature Pyramid Network (FPN) [28] + Path Aggregation Network (PAN) [29] structure. Through the fusion of semantic information and location information, the generalization and expression ability of the model are enriched, and then the feature information is passed to the prediction layer. The three Detects adopted in the Head part, using a grid-based anchor for detection on feature maps of different scales, generated the bounding box and predicted the class. The structure of the YOLOv5 network is shows in Figure 1.

## 3. The Proposed Algorithm

### 3.1. A More Focused and Stable CSR Module

The deepening of the number of network layers will bring the improvement of accuracy. At the same time, the phenomenon of gradient disappearance or a gradient explosion can also occur. The concept of a residual network was introduced in this module, adding the standard residual structure [30]. Through the cross-layer connection structure and lossless propagation gradients, we effectively avoided the gradient disappearance and gradient explosion, and ensured the stability of the model. Meanwhile, this module adds the SimAM [31] attention mechanism. At present, attention mechanisms can be roughly divided into channel attention (traditional one-dimensional attention) and spatial attention (traditional two-dimensional attention). One-dimensional and two-dimensional attention may limit their ability to learn and discern cues. While SimAM attention is three-dimensional attention, it can evaluate the importance of each neuron and infer the 3D attention weight of the feature map in a layer, which guarantees the important feature information to pay attention to. At the same time, the attention mechanism does not need to add parameters to the original network, which ensures a reasonable running speed when paying better attention to the target features.

The input image feature information is transmitted in two ways: a and b. Path a is a standard residual structure, and because of the existence of this branch, the loss can directly transmit the gradient to the previous network through this shortcut when the network back-propagates, which effectively alleviates the gradient disappearance and gradient explosion. Path b first goes through a convolution module, and then it is divided into two paths, c and d; c passes through a convolution module, d passes through the BottleNeck1 module and a convolution module. After, the two paths c and d are Concat into path b. Then, the features of a and b are connected. The total feature information undergoes a convolution module and then receives the weight distribution of SimAM attention, so as to pay attention to the links that need more attention.

**Figure 2:**
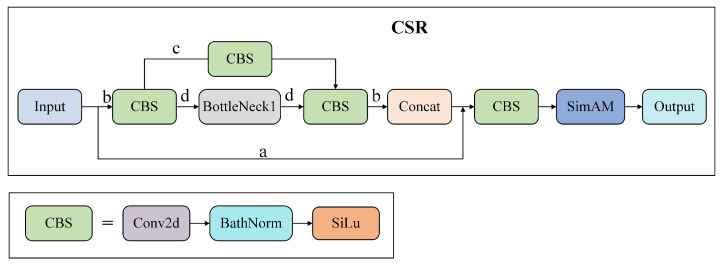
CSR module network structure diagram.

CSR modules (C stands for convolution module, which mainly performs feature extraction. S stands for SimAM [31] attention mechanism, which mainly focuses on important feature information for performance optimization. R stands for Resnet architecture [30], which mainly performs gradient optimization) are deployed in the Backbone part of YOLOv5s and replaced four C3 modules in the Backbone network. At the initial stage of feature extraction, it always pays attention to effective information to ensure the transmission of useful information, prevent gradient disappearance or gradient explosion, and alleviate network degradation. Figure 2 shows the CSR module network structure diagram.

### 3.2. More Accurate Small Object Detection Mechanism (MASD)

Based on the definition of absolute scale and the actual application of UAV shooting scenes, the YOLOv5s algorithm finds it difficult to capture small targets with low resolution due to its large down-sampling multiple, deep feature layer, small detection head size, and large anchor box [32]. The detect head sizes of the YOLOv5s algorithm are 20 × 20, 40 × 40, and 80 × 80. Taking the 20 × 20 detection head as an example, dividing the length and width of the feature map into 20 equal parts will make it easier to detect and frame large targets. Therefore, the feature information of the large target will be extracted completely, and the ability to capture information of the small target object with low resolution will be poor. YOLOv5s selects three anchor boxes [32] with different aspect ratios to frame the target. After many experiments, these three kinds of anchor boxes will increase a lot of redundant useless information in the process of taking small target boxes. The aforementioned issues could lead to weak detection performance of the original algorithm for small targets.

**Figure 3:**
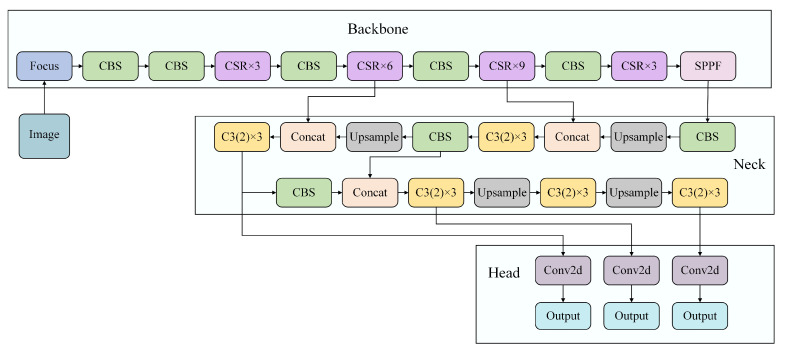
Diagram of the detection head network structure.

In the UN-YOLOv5s algorithm, the MASD mechanism removed the concept of large target detection and focuses on the detection of small and medium targets in UAV shooting. The 20 × 20 detection heads were removed in the proposed method, The size of the detection head approached 160 × 160 by combining up-sampling and the C3 module, increasing the scale of the detection head while extracting target features stably. In this way, the feature map grid will get a more detailed distinction, and the feature extraction for small targets will be more targeted. Therefore, the detection effect of small target objects will be significantly enhanced. The final sizes of UN-YOLOv5s detection heads are 40 × 40, 80 × 80, and 160 × 160. Not only that, we changed the anchor size in the original algorithm. It is more effective to take small targets, successfully removing most of the redundant information, and is more suitable for small target detection in aerial photography. Figure 3 represents the diagram of the detection head network structure.

### 3.3. Multi-Scale Feature Fusion Path (MCF)

In the process of a convolutional neural network [33], the deeper the network layer, the stronger the semantic information of the target, and the better the prediction effect of the model on the target. However, at the same time, the location information of the target will be weaker and weaker. The location information of small targets will be lost. During the process of continuous convolution, it is easy to miss the location information of small targets [34]. So, shallower location information is needed for small targets detection.

**Figure 4:**
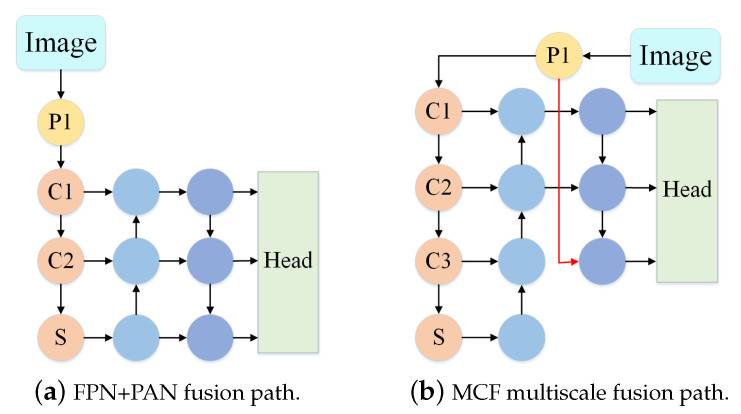
Comparison map of feature fusion paths.

The MCF path of the UN-YOLOv5s algorithm fused the information of different layers and extracted the deep semantic information while introducing the shallow location information. The algorithm used FPN [28] to up-sample from top to bottom, transferring semantic information from deep layers to shallow layers to enhance semantic representation at multiple scales. Through bottom-up down-sampling, the location information of shallow layers was transmitted to deep layers to increase the localization ability at multiple scales. At the same time, adding the shallower and more complete feature information in the Backbone network, it ensured the prediction accuracy of the model for medium and large targets while improving the ability to detect small targets. FPN [28] and improved down-sampling based on PAN [29] were used for feature fusion. Improving the semantic information and location information in the feature map makes the model better and more generalized. Accurate prediction of objects of different sizes is guaranteed. Figure 4 presents the comparison graph of feature fusion paths. Where (a) represents the feature fusion path of FPN [28] and PAN [29], where (b) represents the MCF feature fusion path.

**Figure 5:**
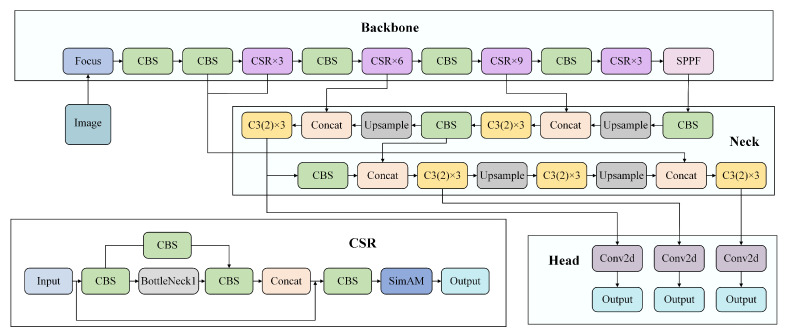
Network structure diagram of UN-YOLOv5s.

Based on the above three innovations, the construction of UN-YOLOv5s is completed. It effectively improved the detection performance of the original algorithm for aerial images. Using the CSR module, implementing gradient optimization to ensure the stable transmission of information, the weight distribution of important factors is completed. Using the MASD mechanism, the accurate positioning of small target detection is realized and redundant information is removed to ensure the effectiveness of the information. Using the MCF path, by fusing shallow location information with deep semantic information, the richness of the feature information is improved. Figure 5 shows the network structure diagram of UN-YOLOv5s.

## 4. Experimental Results

The UN-YOLOv5s and YOLOv5s algorithms were compared on the VisDrone official dataset [35] and the performance of both was evaluated. The VisDrone dataset was collected by the AISKYEYE team, Machine Learning and Data Mining Lab, Tianjin University, designed to facilitate visualization of (remote) data, with a focus on supporting scientific experiments. The benchmark dataset consists of 288 video clips, consisting of 261,908 frames and 10,209 still images, captured by various UAVs. The dataset is rich in content, including targets in different environments, different locations, different weather, and day and night. This dataset has 10 predefined categories, such as pedestrian, car, motorcycle, bus, etc. The rich scene and enough categories are enough for us to deploy the UAV to perform specific aerial surveillance functions.

The experiment was based on a pytorch framework [36] and programmed with python language. The hardware configuration for this experiment are as follows: an i7-12700H processor, 16G RAM, and an Nvidia GeForce RTX3060 (6GB) graphics card. During training, the size of the input image is uniformly adjusted to 640 × 640.

### 4.1. Loss Function Comparison

**Figure 6:**
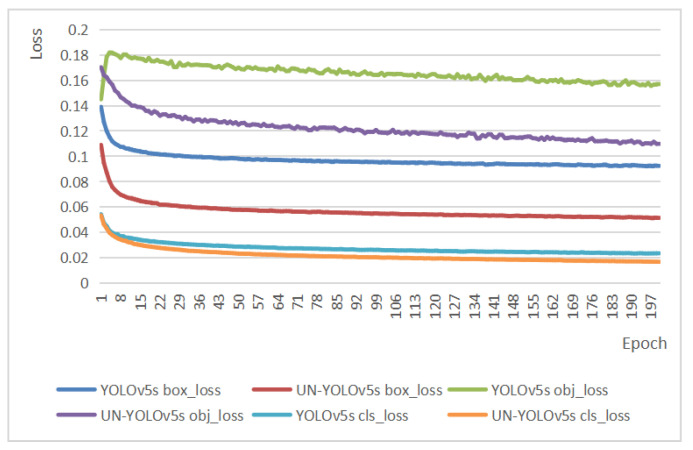
Loss function comparison.

Figure 6 represents the comparison between YOLOv5s and UN-YOLOv5s in terms of classification loss, localization loss, and confidence loss. The smaller the loss function [27], the smaller the difference between the predicted value and the true value. The model adds a SimAM [31] attention mechanism and standard residual structure. It not only improves the mAP but also reduces the risk of overfitting to a certain extent. The experimental results showed that, whether in box-loss, obj-loss, or cls-loss, the loss function of UN-YOLOv5s is lower, and its classification effect, positioning effect, and confidence effect are better. This shows its good prediction performance and stability.

### 4.2. Experimental Results of UN-YOLOv5s

**Figure 7:**
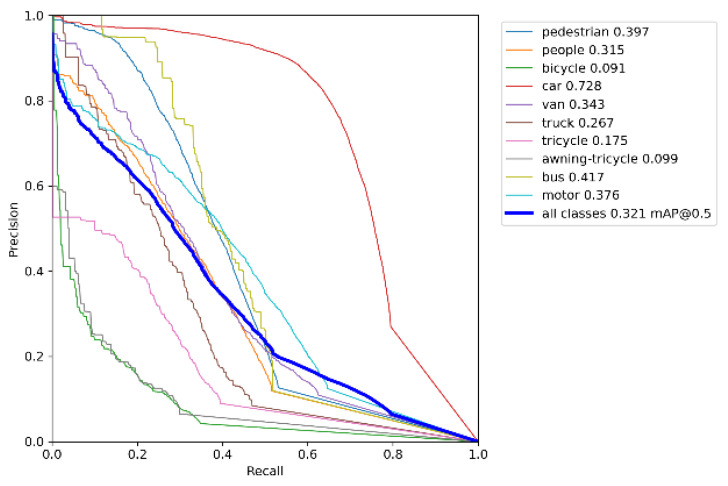
PR-curve for YOLOv5s.

**Figure 8:**
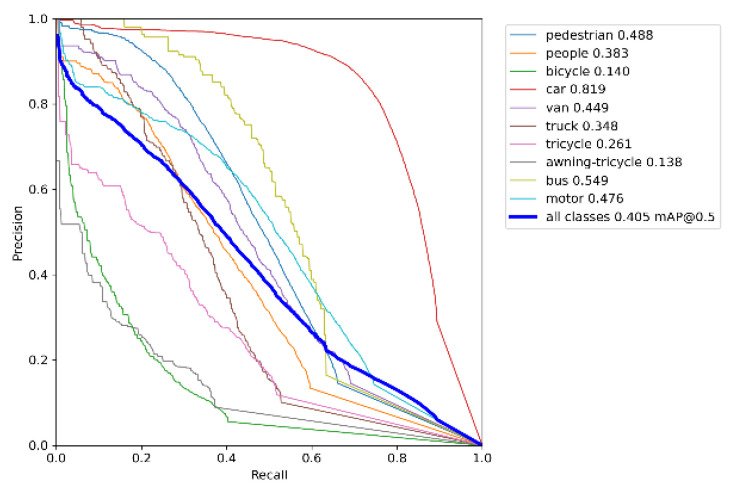
PR-curve for UN-YOLOv5s.

Figure 7 demonstrates the PR-curve [37] performance of YOLOv5s on the VisDrone dataset [32]. Figure 8 illustrates the PR-curve performance of UN-YOLOv5s on the VisDrone dataset [32]. P stands for precision, R stands for recall. The area that the PR-curve contains along the P and R axes is AP. The average of AP values for all categories is mAP. The experimental results showed that, after improvement, the UN-YOLOv5s algorithm improved the mAP@0.5 by 8.4% on all classes. The AP of pedestrian, car, van, truck, tricycle and bus are all increased by more than 8%. Bus has the highest accuracy improvement of 13.2%. At the same time, the prediction accuracy of other categories of targets also increased. Not only that, it can be seen from the figure that the UN-YOLOv5s curve is smoother, more continuous, and stable. The PR curve is sensitive to data imbalance. The change of the ratio of positive and negative samples will cause a large change in the PR-curve. The improved algorithm has higher accuracy, makes the ratio of positive and negative samples more balanced, and has strong robustness. The UN-YOLOv5s algorithm presented a more comprehensive and powerful detection ability.

### 4.3. Example Effect Diagram

**Figure 9:**
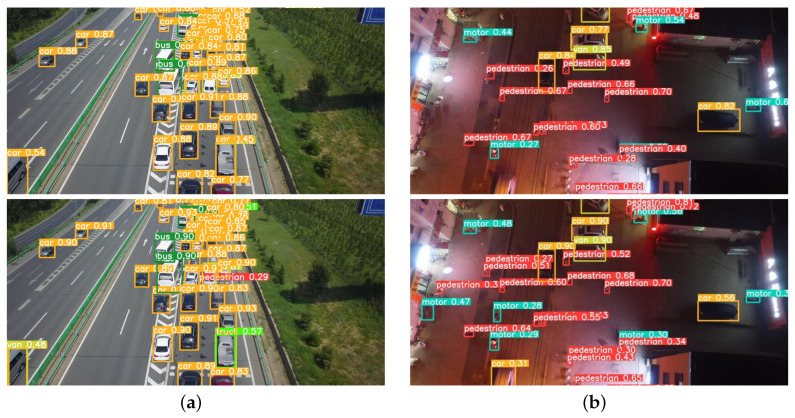
Effect comparison in complex road scenes.

**Figure 10:**
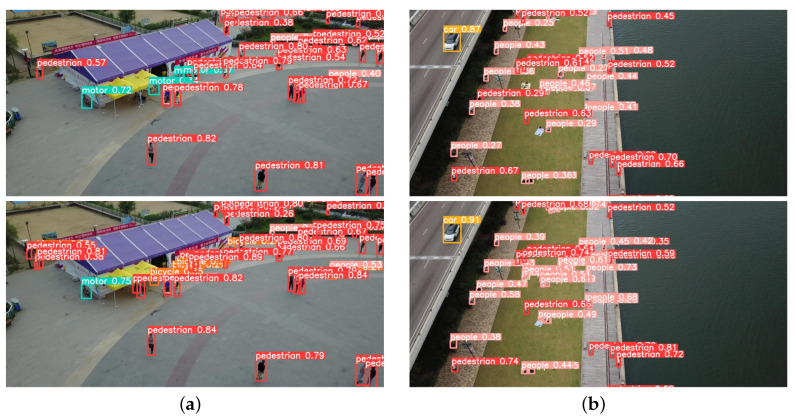
Effect comparison in entertainment and leisure scenes.

Figure 9 shows the effect comparison in complex road scenes on the VisDrone dataset [32]. Figure 10 shows the effect comparison in entertainment and leisure scenes on the VisDrone dataset [32]. Through comparison, it can be seen that whether it is a square, a highway, a block road at night, or a play place by water, UN-YOLOv5s has better performance ability. It can detect objects in denser areas, objects in more remote areas, and objects in more corner areas. At the same time, it is more accurate to divide and identify. As shows in Figure 9a, YOLOv5s cannot detect the pedestrian getting off the bus, recognize a truck accurately, and even misses the vehicles in dense areas. In Figure 9b, YOLOv5s did not detect the passing vehicle and the parked vehicle on the side of the road. At the same time, some people with few Features are not detected. In Figure 10a, YOLOv5s does not detect people in remote places, and could detect a bicycle as a motor. In Figure 10b, the crowd in the dense area is also ignored by YOLOv5s. However, the proposed UN-YOLOv5s algorithm can accurately detect it, and the accuracy of the recognition point of a single object is better than YOLOv5s.

The UN-YOLOv5s algorithm can effectively detect targets in remote places in the picture, monitored people in dense areas, reflected traffic data on crowded roads, reflected driving safety data at night, and reflected regional play data. Not only that, the detection capability of more scenes has also been greatly upgraded, which will not be introduced in detail here. Deployed on UAVs, it is convenient for managers to take safety measures and deal with accidents, which is of great engineering significance.

### 4.4. Ablation Experiments

**Figure 11:**
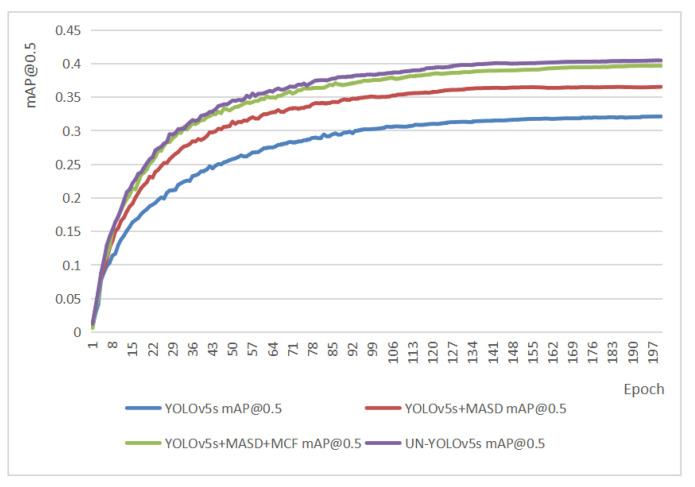
Accuracy comparison of ablation experiments.

This part describes the improvement effect of each improvement module on the basis of YOLOv5s. Table 1 shows the improvement effect in various indicators after the improvement modules were added. Figure 11 represents the mAP@0.5 improvement effect after the improvement modules were added. After the MASD mechanism was added, the recall rate of the model was increased by 4.6%. That is a 3.1% increased in Recall. The mAP@0.5 is upped 4.5% and the mAP@0.5:0.95 improved by 3%. After the MASD mechanism and MCF path were added, the Recall increased by 8%, the Recall increased by 5.4%, the mAP@0.5 improved by 7.6%, and the mAP@0.5:0.95 increased by 4.8%. After the MASD mechanism was added, the MCF path and the CSR module were added. On the basis of stabilizing information dissemination and ensuring network security, the recall rate is successfully increased by 8.2%. That is a 6.2% increase in recall, the mAP@0.5 improved by 8.4%, and the mAP@0.5:0.95 increased by 5.5%.

### 4.5. Comparison of Different YOLO Versions

**Figure 12:**
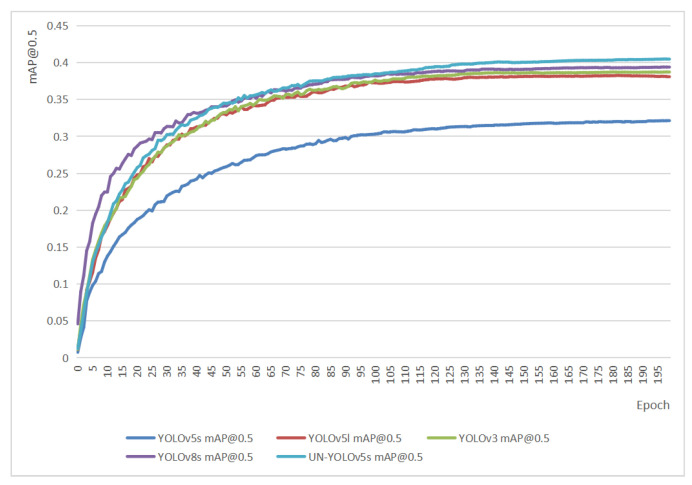
Comparison of mAP@0.5 between UN-YOLOv5s and other versions of YOLO.

Table 2 demonstrates the comparison between UN-YOLOv5s and other versions of YOLO algorithms in various indicators. Figure 12 shows the comparison between UN-YOLOv5s and other versions of YOLO on the most important metric, mAP@0.5. UN-YOLOv5s not only showed its advantages in comparison with YOLOv5s, but also showed its edge in comparison with YOLOv3, YOLOv5l, and YOLOv8s. It achieved the highest accuracy of 40.5%. Table 2 exhibits that within the acceptable range of the GFLOPs improvement, the UN-YOLOv5s algorithm has achieved the first place in recall rate and mAP0.5. Compared with other algorithms, it is more suitable for the deployment of unmanned aerial vehicles.

The mAP@0.5 accuracy of UN-YOLOv5s is 8.4% higher than that of YOLOv5s. It is 2.2% higher than YOLOv5l. It is 1.8% better than YOLOv3, It is 1.1% higher than YOLOv8s. It is worth mentioning that the GFLOPs of YOLOv3 algorithm is 154.7. YOLOv5l has a GFLOPs of 107.8. However, the GFLOPs of UN-YOLOv5s is only 37.4. Compared with the YOLOv3 algorithm, it is reduced by 75.8%. Compared with YOLOv5l, it is reduced by 65.3%. While the accuracy improved, the running speed is also guaranteed. Regarding the UN-YOLOv5s algorithm, the performance is much better than YOLOv3, YOLOv5s, YOLOv5l, and even better than YOLOv8s.

## 5. Discussion and Future Research

A UAV-based detection algorithm, while ensuring the improvement of detection accuracy, is necessary to ensure the real-time performance of detection, validity, and ease of deployment. This algorithm has high detection accuracy. The theoretical basis and practical effect are relatively mature, easy to deploy, and have good versatility. On this basis, by constantly understanding the data of aerial images, it is evident that small and medium targets account for the vast majority of aerial images. The detection head and anchor box adapt the UAV to capture medium and small targets. By fused paths of more scales, the target feature information and location information are mined, and the target features are more accurately locked. The CSR module is added to stabilize information dissemination and focus on important information factors. Finally, the mAP of the proposed algorithm is improved by 8.4% compared with the original algorithm in the VisDrone dataset [32]. We are not only concerned with the comparison of the improved algorithms. Compared with YOLOv5l, a larger model with the same YOLOv5 version, and compared with the same series YOLOv3 and YOLOv8s, the mAP of UN-YOLOv5s is the highest. It even reduced the amount of calculation by 65.3% compared with YOLOv5l. It is 75.8% less than YOLOv3. Through a large number of experimental data comparison and an example diagram display, UN-YOLOv5s is much better than YOLOv5s. It is more stable, precise, and expressive. In future research, we will be striving to promote the deployment and application of UN-YOLOv5s. At the same time, we will keep thinking about the advantages and limitations of this algorithm, trying to improve the accuracy and speed of detection, and overcome the problem of software and hardware adaptation. We will strive to obtain a major breakthrough in this field.

## Figures and Tables

**Table 1:** Comparison of ablation experiment indicators.

Mehods	mAP@0.5(%)	mAP@0.5:0.95(%)	P	R	GFLOPs	Speed (ms)
YOLOv5s	32.1	17	0.407	0.342	15.8	7.4
YOLOv5s+MASD	36.6	20	0.453	0.373	33.9	9.4
YOLOv5s+MASD+MCF	39.7	21.8	0.487	0.396	35.6	9.1
UN-YOLOv5s	40.5	22.5	0.489	0.404	37.4	9.1

**Table 2:** Comparison of ablation experiment indicators.

Mehods	mAP@0.5(%)	mAP@0.5:0.95(%)	P	R	GFLOPs	Speed (ms)
YOLOv5s	32.1	17	0.407	0.342	15.8	7.4
YOLOv5l	38.3	21.5	0.499	0.382	107.8	15.4
YOLOv3	38.7	21.2	0.492	0.383	154.7	19.3
YOLOv8s	39.4	23.5	0.504	0.385	28.5	5.6
UN-YOLOv5s	40.5	22.5	0.489	0.404	37.4	9.1

## Data Availability

All data in this study are included in this paper.

## Sample Availability

The source code for the experiments is available from the corresponding author.
